# Supplementary material for: Effectiveness of Extended or Continuous vs. Bolus Infusion of Broad-Spectrum Beta-Lactam Antibiotics for Febrile Neutropenia: A Systematic Review and Meta-Analysis
Source: Antibiotics (Basel). 2023 Jun 7;12(6):1024. doi: 10.3390/antibiotics12061024 (PMC10295281; doi:10.3390/antibiotics12061024)
Supplement: Supplementary file 1 [file antibiotics-12-01024-s001.zip › antibiotics-2372236-supplementary.pdf]

Figure S1. Identification process for eligible studies.

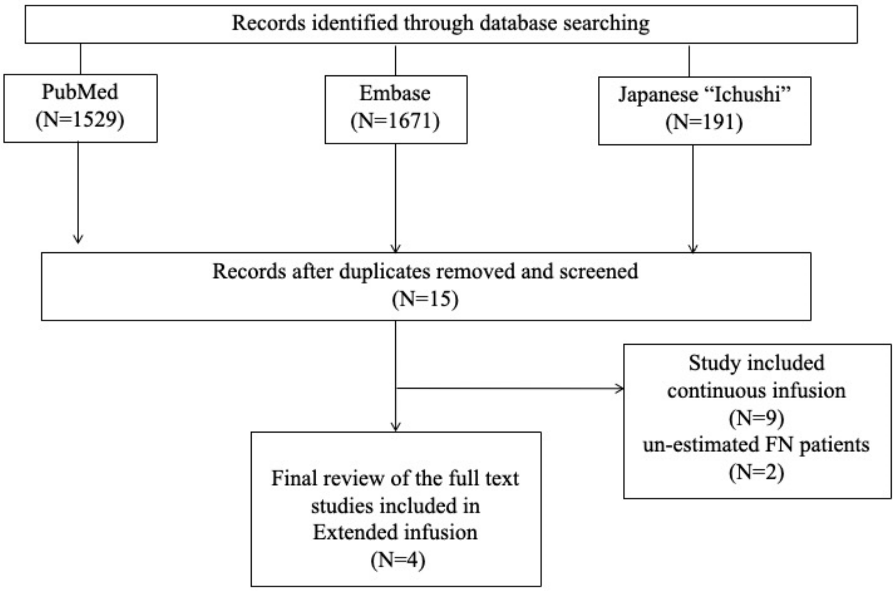

Figure S2. Summary of risk of bias in two randomized controlled trials and two retrospective studies.

RCT	Randomization sequence generation	Allocation concealment	Blinding of participant and clinician	Blinding of outcome assesment	Imcomplete outcome data	Selective reporting	others
Ron 2018	low	low	high	low	low	unclear	low
Rebekah 2018	low	unclear	unclear	unclear	unclear	unclear	low
Retrospective	Selection of participants	Confunding variables	Measurment of exposure	Blinding of outcome assesment	Imcomplete outcome data	Selective outcome reporting	
Crawford 2022	low	high	low	high	low	low	
Csaba 2014	low	high	low	high	low	low	

The risk of bias included randomization sequence, concealment, blinding of participants and clinicians, incomplete outcome data, selective reporting, and others in RCT. The risk of bias included selection of participants, confounding variables, measurement of exposure, blinding of outcome assessment, incomplete outcome data, and selective outcome reporting in the retrospective study.
Green; low risk of bias, yellow; unclear risk of bias, red; high risk of bias. [15–18]

Table S1. Characteristics of the included randomized controlled trials and retrospective studies.

Articles	Published country	Study design	Patient characteristics/ Type of beta-lactam antibiotics	Intervention	Comparison
Crawford 2022 [18]	USA	retrospective	Hematologic malignancy BI:CFPM 2 g q8h, EI:CFPM 1 g q8h	EI (n = 98) CFPM for 4 h	BI (n = 95) CFPM for 30 min
Ron 2018 [15]	Israel	RCT	Hematologic malignancy PIPC/TAZ 4.5 g q6h, CAZ 2 g q8h (if penicillin allergy) Excluded if outpatient, maintenance therapy for ALL, CrCl <40 mL/min, colonization of PIPC/TAZ or CAZ resistant pathogen	EI ITT (n = 47), PP (n = 43) PIPC/TAZ, CAZ 4 h	BI ITT (n = 58), PP (n = 48) PIPC/TAZ, CAZ 30 min
Rebekah 2018 [17]	USA	RCT	Hematologic malignancy CFPM 2 g q8h Excluded if cephalosporin allergy, CrCl <50 mL/min, concurrent infusion of gram-negative effective antibiotics, sepsis, and solid	EI (n = 30) CFPM 3 h	BI (n = 33) CFPM 30 min
Csaba 2014 [16]	Spain	retrospective	Hematologic malignancy MEPM 1 q q8h Excluded if CrCl <50 mL/min	EI (n = 76) MEPM for 4 h	BI (n = 88) MEPM for 30 min

Abbreviations: BI, bolus infusion; CAZ, ceftazidime; CFPM, cefepime; Crcl, creatinine clearance; EI, extended infusion; ITT, intention-to-treat; MEPM, meropenem; PIPC/TAZ, piperacillin/tazobactam; PP, per-protocol; CR, carbapenem-resistant; FN, febrile neutropenia; MIC, minimal inhibitory concentration; PK/PD, pharmacokinetic/pharmacodynamic; RCT, randomized control trials; Vd, volume of distribution
